# Adherence to Intranasal Corticosteroids in Patients with Severe Asthma and Nasal Polyposis: Pharmacological and Clinical Factors Involved

**DOI:** 10.3390/jcm14145070

**Published:** 2025-07-17

**Authors:** Elena Villamañán, Daniel Laorden, María Enriqueta Ibáñez, Leticia De las Vecillas, Carlos Carpio, Carolina Alfonso, Javier Domínguez-Ortega, David Romero, Santiago Quirce, Rodolfo Álvarez-Sala

**Affiliations:** 1 Pharmacy Department, Hospital Universitario La Paz, 28046 Madrid, Spain; elena.villamanan@salud.madrid.org; 2 Department of Medicine, Universidad Autónoma de Madrid, 28047 Madrid, Spain; carlosjavier.carpio@salud.madrid.org (C.C.); rodolfo.alvarezsala@salud.madrid.org (R.Á.-S.); 3 La Paz University Hospital Research Institute (IdiPAZ), 28046 Madrid, Spain; leticia.vecillas@salud.madrid.org (L.D.l.V.); javier.dominguez@salud.madrid.org (J.D.-O.); davizrom@hotmail.com (D.R.); santiago.quirce@salud.madrid.org (S.Q.); 4 Pulmonology Department, Hospital Universitario La Paz, 28046 Madrid, Spain; 5 Allergy Department, Hospital Universitario La Paz, 28046 Madrid, Spain; 6 Otorhinolaryngology (ENT) Department, Hospital Universitario La Paz, 28046 Madrid, Spain; carolina.alfonso@salud.madrid.org; 7 Surgical Deparment, Universidad Autónoma de Madrid, 28047 Madrid, Spain

**Keywords:** nasal polyposis, severe asthma, intranasal corticosteroids, adherence

## Abstract

**Background/Objectives:** Patients with severe asthma (SA) commonly present with coexisting nasal polyposis (NP), often requiring treatment with intranasal corticosteroids (INC). However, adherence to INC in this population remains inadequately characterized despite its clinical significance. This study aimed to evaluate adherence to INC in patients with SA and NP and to identify clinical and pharmacological factors associated with adherence levels. **Methods:** We conducted a retrospective observational study including adult patients with SA and NP treated with INC and followed at a tertiary asthma unit in Madrid during 2024. Adherence was assessed via medication possession ratio (MPR) over six months, with poor adherence defined as MPR < 50%. Pharmacological, clinical and demographic variables were analyzed for associations with adherence. **Results:** Of the 188 patients evaluated, 86 (45.7%) were prescribed INC. Poor adherence was observed in 53.5% of these patients. Women exhibited significantly lower adherence compared to men (*p* < 0.05). Fluticasone was the most commonly prescribed INC (54.6%), with no significant adherence differences across corticosteroid types. Patients on maintenance systemic corticosteroids had higher adherence (85.7%, *p* < 0.05), whereas those receiving biologic therapies tended toward lower adherence (51% poor adherence), though this was not statistically significant. Higher adherence was associated with increased disease severity, as indicated by multiple endoscopic sinus surgeries (*p* < 0.05). No significant differences were observed in spirometry or Asthma Control Test scores. **Conclusions:** Adherence to INC in patients with SA and NP is suboptimal, particularly among women and patients on biologics. Greater disease severity correlates with improved adherence. Targeted interventions are necessary to enhance adherence and optimize disease management in this population.

## 1. Introduction

The respiratory system, though anatomically divided into upper and lower tracts, operates as an integrated functional unit. Numerous studies have revealed shared inflammatory mechanisms underlying diseases that affect both tracts, such as asthma and chronic rhinosinusitis (CRS), with or without nasal polyposis (NP). Patients with CRS are 3.5 times more likely to develop asthma. Moreover, individuals with CRS and nasal polyposis (CRSwNP) who also have comorbid asthma tend to experience more severe disease, including higher recurrence rates of NP. Asthma in patients with NP is also more difficult to control and is associated with greater corticosteroid dependence than asthma alone (4% vs. 1%) [1,2].

The current pharmacological management of NP relies heavily on the continuous and prolonged use of high-dose intranasal corticosteroids (INC) [3,4,5]. Despite this, up to 40% of patients report inadequate disease control, often necessitating endoscopic sinus surgery (ESS) [6,7,8]. Post-surgical maintenance therapy with INC remains essential to prevent recurrence and improve outcomes [9].

Over the past decade, biologics have become increasingly common in the treatment of severe asthma and, more recently, in the management of NP. Biologics such as omalizumab (anti-IgE) [10,11], mepolizumab [12], reslizumab (anti-IL-5) [13] and dupilumab (anti-IL4Rα) have demonstrated efficacy in reducing NP size, improving nasal symptoms (including smell), and enhancing quality of life. Mepolizumab and dupilumab [12,14,15,16] in particular, have shown modest reductions in surgical intervention needs. Moreover, tezepelumab has been proven to reduce nasal polyp size and the need for surgery or systemic glucocorticoids compared to placebo in adults with severe, uncontrolled (CRSwNP) [17]. Nevertheless, despite these promising outcomes, INC therapy remains a critical component of disease control in NP patients.

Adherence to INC therapy, however, is well-documented to be suboptimal [18,19,20,21]. Valverde-Monje et al. [22] reported that only 43% of patients with asthma and NP adhered to prescribed INC regimens, with overall adherence rates falling below 50%. A review by Norelli et al. [23] highlighted that the majority of CRSwNP studies involving biologics did not assess INC adherence. Among 17 studies reviewed—including randomized controlled trials and real-world investigations—only three examined adherence, and just one explored factors contributing to poor adherence. This lack of data presents a potential bias in interpreting the effectiveness of biologic treatments.

Although INC adherence has been broadly studied [21,23], to our knowledge, no studies have specifically assessed it in patients with both severe asthma (SA) and NP. Many of these patients are prescribed biologics for SA, which also benefit NP. It is plausible that those receiving biologics might demonstrate even lower INC adherence than those not on biologics.

This gap in the literature prompted our study. Our objective was to evaluate adherence to INC therapy in patients with SA and NP and to identify adherence differences based on clinical and pharmacological characteristics. The aim was to pinpoint potential areas for intervention to optimize the management of this complex patient population.

## 2. Materials and Methods

This retrospective observational study included patients diagnosed with severe asthma. Participants were managed by a multidisciplinary team at an asthma clinic within a tertiary care hospital in Madrid, Spain. The study was conducted between January and December 2024. Ethical approval was obtained from the Institutional Review Board of La Paz University Hospital (IRB protocol No. PI-6272). As this was an observational study, informed consent was not required.

These patients typically exhibited uncontrolled asthma despite high-dose inhaled corticosteroids combined with long-acting β_2_-agonists or oral corticosteroids (GINA Step 5 [24] and GEMA Step 6 guidelines [25]. Many of them are under biologic therapy based on guideline indications.

The proportion of patients demonstrating poor adherence to INC therapy was assessed as the primary outcome. Adherence was assessed using pharmacy dispensing data through the medication possession ratio (MPR), using the following formula:

MPR = (total number of INC units dispensed ÷ total number of INC units prescribed) × 100

Prescription and pharmacy refill data from the previous six months were analyzed. Based on the previously reported low adherence rates in patients with severe asthma, a threshold of >50% MPR was considered indicative of acceptable adherence, in line with the existing literature [21,22,26,27,28].

Secondary outcomes included adherence differences according to demographic characteristics, pharmacological variables (type of INC, use of biologics, and need for systemic corticosteroids), and clinical variables such as presence of asthma (patients requiring high doses of inhaled corticosteroids), allergic rhinitis (IgE-mediated inflammatory disorder of the nasal mucosa triggered by exposure to environmental allergens), the presence and severity of nasal polyposis according to international guidelines [29], prior endoscopic sinus surgeries, respiratory function, and comorbidities.

Inclusion criteria for the enrolled patients were the total patients with intranasal corticosteroids (INC) that were evaluated in our severe asthma clinic during 2024.

Data were collected from electronic health records and pharmacy refill systems.

Quantitative variables were expressed as medians and interquartile ranges (IQR), while categorical variables were presented as frequencies and percentages. Comparisons of continuous variables were performed using the Mann–Whitney U test, and categorical variables were compared using the chi-square test or Fisher’s exact test. Cohen’s kappa statistic was used to assess concordance. Statistical analysis was conducted using IBM SPSS Statistics version 19.0, with a significance level set at *p* < 0.05.

## 3. Results

### 3.1. Patient Characteristics

Out of 188 patients evaluated by the Severe Asthma Clinic in 2024, a total of 86 patients (45.7%) were prescribed intranasal corticosteroids (INC). Among these, 60 patients (69.8%) were women. The median age was 63 years (interquartile range [IQR]: 57–71 years).

Regarding comorbid conditions associated with severe asthma, 65 of 86 patients (75.6%) presented with at least one related comorbidity. The most frequently reported were gastroesophageal reflux disease (37.8%), allergic rinitis (35.6%), and bronchiectasis (33.3%), similar to other studies performed in our Severe Asthma Clinic [30,31], and no patients had cystic fibrosis or primary ciliary dyskinesia. Additionally, the vast majority of patients (93.3%) were non-smokers. A detailed summary of patient characteristics is presented in Table 1**.**

### 3.2. Adherence to Intranasal Corticosteroids

Among the 86 patients prescribed intranasal corticosteroids (INC), 46 (53.5%) demonstrated poor adherence, defined as a medication possession ratio (MPR) of less than 50%.

When stratified by sex, a significant difference in adherence was observed: female patients exhibited significantly lower adherence to INC therapy compared to male patients (*p* < 0.05). These results are summarized in Table 2.

### 3.3. Pharmacological Variables and Adherence

Among patients prescribed intranasal corticosteroids (INC), fluticasone was the most frequently prescribed agent, used by 47 of 86 patients (54.6%). In this group, the distribution of adherence was roughly balanced between acceptable and poor. In contrast, adherence to mometasone was notably poorer (Table 3).

Additionally, long-term systemic corticosteroids were used by 7 of 86 patients (8.1%). Among these, 6 patients (85.7%) demonstrated acceptable adherence to INC therapy (*p* < 0.05), suggesting a statistically significant association between systemic corticosteroid use and improved INC adherence (Table 3).

### 3.4. Adherence to Inhaled Therapies and Use of Biologics

Adherence to inhaled maintenance therapies in patients with severe asthma (SA) was generally high. Among those with nasal polyposis (NP), 64 of 86 patients (74.4%) demonstrated acceptable adherence (MPR > 50%). This rate was comparable to that observed in SA patients without NP, 78 of 102 patients (76.5%), indicating no significant difference in adherence between the two groups.

In contrast, overuse of rescue inhalers—defined as the use of more than three canisters per year—was observed in 31 of 86 patients (36%) with NP, highlighting a subgroup with potentially poor symptom control.

**Figure 1:**
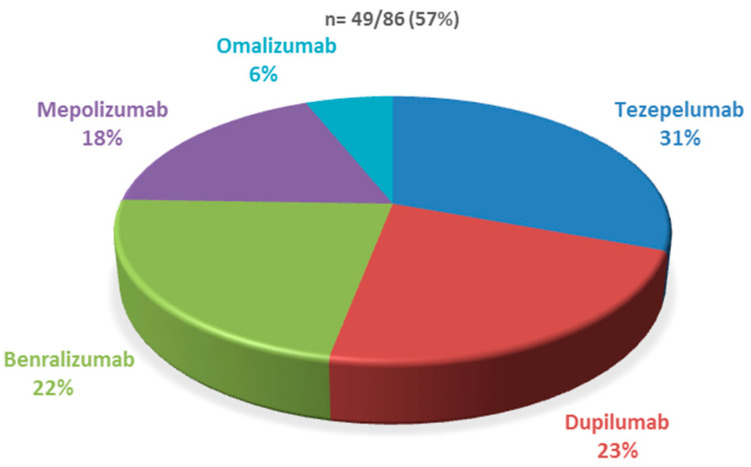
Patients under biologic therapy and intranasal corticosteroids concomitantly.

Regarding biologic therapy, 56.9% of patients with SA and NP were receiving biologic treatment. The most commonly prescribed biologic was tezepelumab, which was used in 30.6% of cases (Figure 1).

### 3.5. Adherence to Intranasal Corticosteroids Among Patients on Biological Therapy

Among patients receiving biologic therapy, 51% demonstrated poor adherence to intranasal corticosteroids (INC), with a medication possession ratio (MPR) below 50%. Although this trend suggests lower adherence among biologic-treated patients, the difference was not statistically significant (*p* = 0.397).

When analyzing adherence by specific biologic agents, patients treated with dupilumab and tezepelumab were more likely to exhibit acceptable adherence to INC. In contrast, patients receiving benralizumab, mepolizumab, and omalizumab showed higher rates of poor adherence compared to acceptable adherence (Table 4).

### 3.6. Adherence and Endoscopic Sinus Surgery

Patients who underwent endoscopic sinus surgery (ESS) for actively severe symptoms demonstrated significantly higher adherence to intranasal corticosteroids, with adherence increasing alongside the number of surgeries performed (*p* < 0.05) (Table 5).

### 3.7. Spirometry and Asthma Control

No statistically significant differences were observed in spirometry parameters or Asthma Control Test (ACT) scores between the groups analyzed (Table 6).

## 4. Discussion

According to our results, there is a high proportion of patients with severe asthma and NP associated needing INC (45.7%). In alignment with previous studies [32], we found a high prevalence of upper airway comorbidities, particularly allergic rhinitis and nasal polyps, among patients with severe asthma.

A key observation was the low adherence to INC, with 53.5% of patients demonstrating poor adherence (MPR < 50%). This is consistent with prior studies reporting suboptimal adherence to intranasal therapies, especially among patients with chronic rhinosinusitis or allergic rhinitis [22].

This finding underscores the importance of addressing treatment adherence in this population, as low adherence can compromise disease control and increase the need for more invasive interventions, such as surgery.

Regarding pharmacological variables, we observed that fluticasone was the most prescribed INC (54.6% of patients), reflecting its widespread use in managing NP. However, no significant differences in adherence were found among different types of INC, suggesting that the choice of INC is not a determining factor in adherence. This finding is consistent with previous systematic reviews that have found no conclusive evidence that one type of INC is superior to another in terms of efficacy [32]. Although the difference was not statistically significant, the previous literature suggests that factors such as dosing frequency, device usability, sensory perception (e.g., taste or nasal irritation), and patient preference may influence adherence [33].

On the other hand, patients receiving maintenance systemic corticosteroids showed higher adherence to INC (85.7% with adherence >50%, *p* < 0.05). This could be explained by the fact that these patients have worse symptom control, leading them to be more adherent to intranasal treatments [34]. In contrast, patients receiving short-course systemic corticosteroids showed significantly lower adherence (*p* < 0.05), suggesting that improving INC adherence in this population could reduce the need for systemic corticosteroids.

Notably, female patients showed lower adherence rates, consistent with existing evidence linking female sex to reduced medication adherence across various chronic illnesses, a pattern that has been described in other chronic diseases and has been linked to gender-based differences in treatment perception, side-effect concerns, and medication beliefs [35]. Also, sociocultural factors, including caregiving responsibilities and healthcare engagement patterns, may contribute to this disparity. Although broad meta-analyses report no overall sex differences, subgroup analyses suggest that older women may be particularly vulnerable to poor adherence, emphasizing the need for gender-sensitive patient education strategies [36,37,38].

As expected, taking into account the benefits for NP of biologics used for SA [39], patients receiving biologic therapies tended to exhibit poorer adherence to INC (51% poor adherence), though this trend did not reach statistical significance. This may be explained by symptomatic improvement associated with biologics, potentially leading patients to underestimate the necessity of continued intranasal corticosteroid use. Biologics have demonstrated significant improvements in both lower and upper airway symptoms, including reductions in nasal polyp burden, potentially reinforcing the value of concomitant topical therapy. Although the differences in adherence by biologic type were not statistically significant, this trend warrants further investigation, mainly real-world studies [40].

Furthermore, adherence positively correlated with disease severity, as patients who underwent multiple endoscopic sinus surgeries demonstrated significantly better adherence (*p* < 0.05). This suggests that heightened disease burden and the tangible consequences of recurrence promote greater patient engagement with prescribed therapies. Despite these adherence differences, no significant variations were detected in pulmonary function tests or Asthma Control Test (ACT) scores, suggesting that INC adherence may primarily influence nasal symptomatology rather than overall respiratory function in this cohort.

### Study Limitations

This study has several limitations inherent to its retrospective design, which restricts the ability to establish causality between adherence and clinical outcomes. Adherence was assessed using the medication possession ratio (MPR) derived from pharmacy refill data, which may not accurately capture actual medication usage or patient behavior. Moreover, the association found between systemic corticosteroid use and higher adherence may reflect more severe disease rather than better behavior.

Additionally, the relatively small sample size may have limited statistical power to detect significant differences in certain subgroups, particularly regarding the impact of biologic therapies on adherence. Furthermore, the study was conducted at a single tertiary care center, which may limit the generalizability of the findings to other populations or healthcare settings. Future prospective, multicenter studies employing direct measures of adherence and larger cohorts are warranted to confirm and expand upon these findings.

## 5. Conclusions

Adherence to intranasal corticosteroids among patients with severe asthma and nasal polyposis remains suboptimal, particularly in women and those treated with biologics. Conversely, patients with more severe disease manifestations—such as those requiring multiple surgeries or maintenance systemic corticosteroids—demonstrate higher adherence levels, indicating that disease severity may serve as a motivating factor for treatment compliance.

These findings highlight the need to implement tailored educational and follow-up interventions aimed at improving adherence in this vulnerable population. Focused efforts on high-risk groups could enhance disease control, reduce the need for systemic corticosteroids, and potentially diminish the frequency of surgical interventions, ultimately improving patient outcomes.

## Figures and Tables

**Table 1:** Patient characteristics.

Characteristic	N = 86
Median age	63 (IQR 57−71)
Female	60 (69.8%)
Comorbidity associated with severe asthma	N = 65/86 (75.6%)
Gastroesophageal reflux	17 (37.8%)
Allergic rhinitis	16 (35.6%)
Bronchiectasis	15 (33.3%)
Aspirin-Exacerbated Respiratory Disease	9 (20.0%)
Anxiety/Depression	5 (11.1%)
Allergic bronchopulmonary aspergillosis	3 (6.7%)

**Table 2:** Adherence to intranasal corticosteroids according to patient’s characteristics.

Characteristic	Acceptable Adherence to INC (>50%)	Poor Adherence to INC (<50%)	*p*-Value
Gender			
Females	23 (57.5)	37 (80.4)	0.038
Males	17 (42.5)	9 (19.6)	
Asthma-related comorbidities			
Gastroesophageal reflux	9 (39.1)	8 (36.4)	1.000
Allergic rhinitis	9 (39.1)	7 (31.8)	0.841
Bronchiectasis	10 (43.5)	5 (22.7)	0.246
Aspirin-exacerbated respiratory disease	9 (20)	3 (13.6)	0.502
Anxiety/depression	4 (11.1)	1 (4.5)	0.370
Allergic bronchopulmonary aspergillosis	1 (4.3)	2 (9.1)	0.968

**Table 3:** Adherence to intranasal corticosteroids according to corticotherapy type.

Corticosteroid	Acceptable adherence to INC (>50%)	Poor adherence to INC (<50%)	*p*-Value
Nasal corticosteroid	40	46	0.285
Fluticasone	23 (26.7)	24 (27.9)	
Mometasone	16 (18.6)	20 (23.2)	
Triamcinolona	-	1 (2.2)	
Mometasona + olopatadine	1 (2.5)	**-**	
Concomitant systemic corticosteroids			
Long-term systemic corticosteroids	6 (85.7)	1 (14.3)	0.027
Short courses of systemic corticosteroids	3 (21.4)	11 (78.6)	0.006

**Table 4:** Adherence to intranasal corticosteroids according to biologic therapies.

Biologic Therapy	Acceptable Adherence to INC (>50%)	Poor adherence to INC (<50%)
Dupilumab	7 (29.2)	4 (16)
Tezepelumab	8 (33.3)	7 (28)
Benralizumab	5 (20.8)	6 (24)
Mepolizumab	4 (16.7)	5 (20)
Omalizumab	-	3 (12)

INC: intranasal corticosteroids.

**Table 5:** Adherence to intranasal corticosteroids in patients under biologics according to previous nasal polyposis surgeries.

	Acceptable Adherence to INC (>50%) N = 40	Poor Adherence to INC (<50%) N = 46	*p*-Value
Number of endoscopic sinus surgery	2.00 [1.00, 3.25]	0.50 [0.00, 1.75]	0.042

INC: intranasal corticosteroids.

**Table 6:** Adherence to intranasal corticosteroids according to respiratory function.

Respiratory Outcomes	Acceptable Adherence to INC (>50%)	Poor Adherence to INC (<50%)	*p*-Value
**FEV 1 pre-biologic % ** (median(IQR))	79.00 [66.00, 91.75]	77.00 [72.00, 88.00]	0.704
**FEV 1 mL pre** (median(IQR))	2,01 [1.537, 2.895]	2095 [1.852, 3.307]	0.978
**FVC pre-biologic % ** (median(IQR))	88.50 [77.00, 105.50]	93.00 [87.50, 105.00]	0.244
**FVC ml pre** (median(IQR))	3.005 [2.435, 4.145]	2800 [2.640, 3.790]	1.000
**FEV 1/FVC pre-biologic % ** (median(IQR))	72.02 [64.61, 79.32]	67.87 [60.94, 76.94]	0.109
**FENO pre-biologic % ** (median(IQR))	36.00 [18.50, 57.90]	24.00 [10.82, 45.63]	0.242
**FEV 1 post-biologic % ** (median(IQR))	83.00 [77.00, 94.00]	78.00 [65.75, 92.00]	0.464
**FEV 1 mL post** (median(IQR))	2030 [1960, 3140]	2330 [1.960, 2.615]	0.808
**FVC post-biologic %** (median(IQR))	114.00 [99.00, 115.00]	98.00 [88.75, 104.25]	0.092
**FVC ml post** (median(IQR))	3780 [3.290, 4.100]	3110 [2.820, 3.805]	0.465
**FEV 1/FVC post-biologic %** (median(IQR))	62.00 [60.28, 66.25]	66.22 [61.81, 71.35]	0.558
**FENO post-biologic % ** (median(IQR))	37.00 [20.00, 51.00]	24.50 [15.50, 30.00]	0.380
**ACT pre-biologic % ** (median(IQR))	18.00 [10.00, 20.00]	20.00 [17.00, 21.00]	0.130
**ACT post-biologic % ** (median(IQR))	20.00 [16.50, 22.00]	21.00 [18.75, 24.00]	0.400

## Data Availability

The data presented in this study are available on request from the corresponding author due to data protection law.

## Abbreviations

The following abbreviations are used in this manuscript:

SA	Severe asthma
NP	Nasal polyposis
INC	Intranasal corticosteroids
MPR	Medication possession ratio
CRS	Chronic rhinosinusitis
CRSwNP	Chronic rhinosinusitis with nasal polyposis
IQR	Interquartile ranges
ESS	Endoscopic sinus surgery
ACT	Asthma Control Test
